# Taxonomic Analysis of Oral Microbiome during Orthodontic Treatment

**DOI:** 10.1155/2021/8275181

**Published:** 2021-10-28

**Authors:** Alessandra Campobasso, Eleonora Lo Muzio, Giovanni Battista, Domenico Ciavarella, Vito Crincoli, Lorenzo Lo Muzio

**Affiliations:** ^1^Department of Clinical and Experimental Medicine, University of Foggia, Clinica Odontoiatrica, Via Rovelli 50, Foggia 71122, Italy; ^2^Department of Translational Medicine and for Romagna, School of Orthodontics, University of Ferrara, Via Luigi Borsari 46, Ferrara 44121, Italy; ^3^Department of Basic Medical Sciences, Neurosciences and Sensory Organs, University of Bari, Piazza Giulio Cesare 11, Bari 70100, Italy

## Abstract

**Background:**

Orthodontic appliances induce significant changes in the oral microbiome, but this shift in microbial composition has not been well established by the available evidence yet.

**Objectives:**

To perform a systematic review of existing literature in order to assess the taxonomic microbial changes in orthodontic patients during Fixed Appliance Treatment (FAT) and Clear Aligner Treatment (CAT), using next-generation sequencing (NGS) technique of the bacterial 16S rRNA gene. *Search Methods and Selection Criteria*. The search for articles was carried out in PubMed, including articles published in English until May 2021. They included every human study report potentially relevant to the review. *Data Collection and Analysis*. After duplicate study selection and data extraction procedures according to the PICOS scheme, the methodological quality of the included papers was assessed by the Swedish Council on Technology Assessment in Health Care Criteria for Grading Assessed Studies (SBU) method.

**Results:**

The initial search identified 393 articles, 74 of which were selected by title and abstract. After full-text reading, six articles were selected according to inclusion criteria. The evidence quality for all the studies was moderate.

**Conclusions:**

Orthodontic treatment seems to transiently affect the composition of subgingival microbiome, although not salivary, maintaining a stable microbial diversity. Different results were found in the shift of microbiome between plaque and saliva, depending on the type of orthodontic treatment. This review should be interpreted with some caution because of the number, quality, and heterogeneity of the included studies.

## 1. Introduction

In modern society, orthodontic treatment plays a key role not only in the correction of malocclusion, but also in the improvement of aesthetic appearance [1].

Fixed Appliance Treatment (FAT) has been the most traditional and effective orthodontic therapy for over a hundred years [2], but in the last decades the demand for a more aesthetic and comfortable treatment has significantly increased [1] and a growing number of patients seek Clear Aligners Treatment (CAT) [3]. Unlike the conventional FAT that consists of fixed placement of the orthodontic brackets directly on the tooth surface [1], CAT includes a series of removable plastic aligners covering the entire tooth surface [4]. In the literature, the influence of orthodontic appliances on the local environment [5] has been largely reported: in fact, foreign surfaces, such as multibracket devices or aligners, decrease natural self-cleansing by the saliva and the tongue [1] and represent an ideal substrate for bacterial adhesion that make oral hygiene more difficult [4].

While the treatment effectiveness of both FAT and CAT has proven to be similar [2], several studies showed that CAT was better for periodontal health than FAT and that CAT might be recommended for patients at high risk of developing periodontal diseases [1, 6]. The effects of FAT and CAT on the periodontal health reported by previous literature [1, 6–12] were mainly analyzed through clinical (and not microbiological) evaluations, based on the more easy observable clinical parameters [13], but it is well known that the promotion of oral health or progression towards periodontal disease is critically influenced by the invisible microbiota [14].

The oral microbiome contributes to the local and whole-body health of the host through a dynamic balance [15]. Any substantial change to the local environment may alter the host-microbe equilibrium, increasing the risk of oral disease [15]. The increase of plaque accumulation and the subsequent inflammation of periodontal tissues that occurs in orthodontic patients leads to the potential shift of the bacterial oral ecosystem towards a pathogenic state [16].

This microbial shift is the primary etiology factor of periodontal diseases, such as white spot lesions, caries, gingivitis, and periodontal complications that are the most common side effects of the orthodontic therapies [3]. These oral changes contribute not only to the onset of oral diseases but also increase the risk of severity of several systemic diseases including diabetes, cardiovascular disease, and rheumatoid arthritis [17].

Several researchers have investigated the influence of orthodontic appliance on the number and composition of oral microbiota, but most of these outcomes are limited by the inner limitations of the available techniques [16, 18, 19]. In fact, the oral cavity is a complex habitat for bacterial microflora, harboring over 700 species of bacteria and this abundant microflora could not be investigated with conventional analytical approaches [20]. Previously, the microbiome analysis was difficult and limited because it was based on traditional cultural methods. The culture-dependent techniques do not allow a correct analysis of the oral microbiome in orthodontic patients, as the abundant microbial species could not be isolated and cultured at the same time [20].

In the last decades, the development of traditional biochemical methods (most commonly via PCR) allowed the analysis of the specific microbial expression during FAT or CAT therapy, through molecular amplification techniques [21–27].

Nevertheless, these methods targeted only a limited number of pathogens and did not reflect the shift in the microbial community [26].

Whereas initially the microbiological approach was to detect an association between the presence of specific pathogens and periodontal disease, as with many classical infections due to colonization by one or more species that are not normally found on that site and which are specific to that disease diagnosis [17], over a period of time, it has become apparent that oral diseases are mainly associated with a shift in the oral microbiota departing from the species predominating in health towards a greater abundance of communities comprising taxa that were previously minor components of the microbiome [17].

Currently, the advent of next-generation sequencing (NGS) technologies has enabled the analysis of the entire microbial genetic material of the examined samples (and not only of some species), using 16S rRNA genes as targets for microbial identification [28].

Through bacterial 16S rRNA gene sequencing, the NGS method overcomes the limitations of traditional methods and provides a more sensitive and comprehensive amount of data about microbial diversity (including unculturable bacteria, that represent 35% of the 700 oral species), allowing the taxonomic analysis and comparison of bacterial composition from different samples [29].

Despite the proliferation of available sequencing instruments and the exponential decrease in sequencing costs [30], in the literature, there are still very few studies that use the 16S rRNA gene sequencing technique to analyze the dynamic microbial changes during orthodontic therapy [13, 25, 26, 31].

It was therefore necessary to carry out a literature update about the influence of FAT and CAT on the entire oral microbiome, because it may have a useful implication in the daily clinical practice for the prevention and for the therapeutic choice, especially in the susceptible orthodontic patients [17].

The rationale is to analyze the microbial shift induced by the orthodontic treatment and to evaluate the differences in microbial changes that occur during two different types of treatment (fixed or removable), assessing whether CAT influences the local environment in a similar way to traditional FAT or whether either therapy alters the host-microbiome balance more, increasing the risk of periodontal disease.

Therefore, this systematic review aims at identifying existing studies based on a high-throughput sequencing technology evaluating the microbial changes during orthodontic treatment, in order to answer the focused questions:

- What are the taxonomic changes of oral microbiome during FAT or CAT?
- What are the differences in changes of oral flora between FAT and CAT?
- Can orthodontic appliances induce measurable changes in microbiome from healthy to periodontitis?

## 2. Materials and Methods

This systematic review was performed in compliance with the guidelines of the Preferred Reporting Items for Systematic Reviews and Meta-Analyses (PRISMA) checklist.

The research question of the present systematic review was defined according to the PICOs scheme, as follows:

  -P (population/patients): subjects undergoing orthodontic treatment
  -I (intervention): patients received treatment with fixed appliance or clear aligners
  -C (comparison): subjects treated with FAT and/or CAT and/or not receiving any treatment
  -O (outcome): changes in microbial composition, analyzed through the NGS technique of the bacterial 16S rRNA gene

The inclusion criteria were as follows:

- Prospective original studies on human subjects that evaluated the dynamic microbial changes through NGS method in orthodontic patients, at different stages of treatment
- Studies on orthodontic patients in good general health, with no restrictions in terms of malocclusion or age
- Studies on patients treated with fixed metal brackets or removable clear aligners
- Studies that included clear descriptions of the materials and applied technique

The exclusion criteria for this review were as follows:

- Papers conveying non-human studies included in vitro observations or articles focusing on animal experiments
- Papers not in English language
- Studies on patients with periodontal disease
- Studies without specific descriptions of the materials and applied technique
- Studies on patients with ceramic bracket or lingual bracket

### 2.1. Search Strategy for the Identification of Studies

The search for articles was conducted in PubMed, including articles published in English until April 2021. They included every human studies report featuring the keywords “orthodontic” OR “fixed appliance” OR “fixed orthodontic” OR “bracket” OR “clear aligner” OR “removable aligner” OR “Invisalign” AND “microbiota” OR “microbiome” OR “microbiome” OR “oral microbiota” OR “oral microbioma” OR “oral microbiome OR “microflora” OR “microorganism” OR “microbe”” OR “16S rRNA” OR “16S sequencing” OR “next-generation sequencing.” The reference list and citation list of the included studies and reviews were manually searched as well.

### 2.2. Selection of Studies

Titles identified from literature were screened and selected by two independent authors (A.C. and E.L.M.). Duplicate studies were eliminated. Abstracts were examined; full texts were obtained if additional data were needed for the eligibility criteria. Conflicts were resolved by a third author (L.L.M.).

### 2.3. Data Extraction

Characteristics of included studies (study design, sample size, average age of patients, sample site, orthodontic appliance, sample collection time, collection method, analysis method, taxonomic analysis, and microbial outcomes) were independently extracted by two authors (A.C. and E.L.M.). Missing or unclear information has been directly requested from the authors for further clarifications.

## 3. Methodological Quality Assessment

The methodological quality of the included studies was assessed according to the “Swedish Council on Technology Assessment in Health Care Criteria for Grading Assessed Studies” (SBU) method [32]. Articles were graded into three grades (A, B, and C) of evidence:

- A (high level of evidence): randomized clinical study or prospective study with a well-defined control group, defined diagnosis and endpoints, diagnostic reliability tests, and reproducibility tests described
- B (moderate level of evidence): cohort study or retrospective case series with defined control or reference group, defined diagnosis and endpoints, diagnostic reliability tests, and reproducibility tests described
- C (low level of evidence): large attrition, unclear diagnosis and endpoints, and poorly defined patient material

Based on the score assigned to each study, the review's level of available evidence was scored in four grades (1, 2, 3, and 4):

- 1 (strong evidence): at least two studies assessed with level “A”
- 2 (moderate evidence): one study with level ‘‘A” and at least two studies with level ‘‘B”
- 3 (limited): at least two studies with level ‘‘B”
- 4 (inconclusive): fewer than two studies with level ‘‘B.”

### 3.1. Data Synthesis

Due to the lack of homogeneity in the study setting (study design, sample site, sample collection time, and methods), only a systematic review could be realized, and not a meta-analysis.

## 4. Results

### 4.1. Search Results

**Figure 1:**
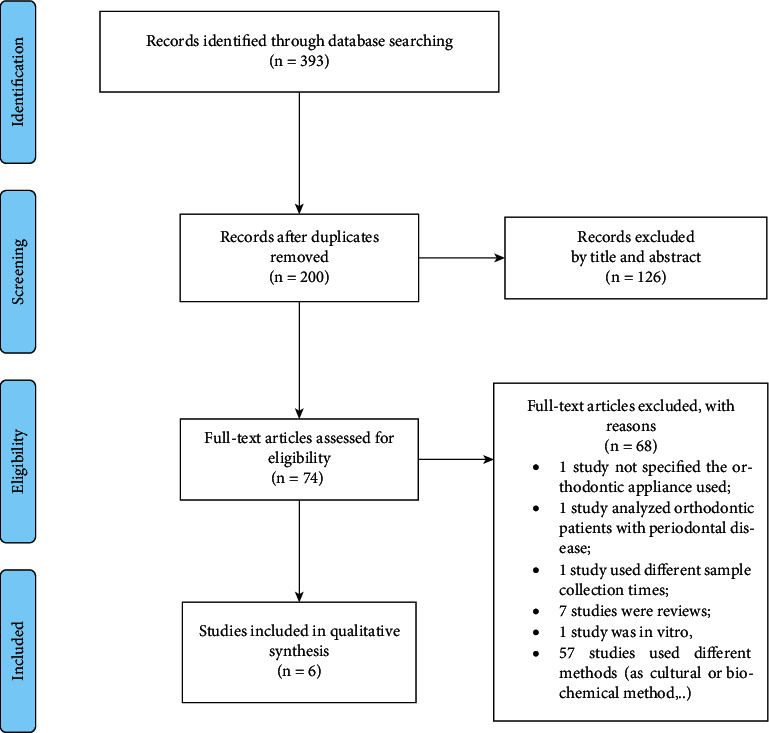
PRISMA flow diagram.

The initial search identified 393 articles from PubMed. After eliminating duplicates and ineligible studies by title and abstract, a total of 74 full texts were screened. Finally, a total of six papers were selected according to eligibility criteria. A manual search was also performed to screen the references of these 6 articles, but no study was included. The flow chart of the eligible studies selection for this review is summarized in Figure 1.

### 4.2. Assessment of Methodological Quality

According to the SBU tool, the quality of evidence for all six studies was moderate (grade B). Thus, the level of evidence for the conclusions of this review was limited (level 3) as well.

### 4.3. Studies Characteristics

The studies characteristics are reported in Table 1.

### 4.4. Studies' Descriptions

Among the included six studies, one study was a randomized clinical controlled trial and five were prospective non-randomized [13, 28, 29, 34, 35]; [3]. Three studies [13, 28, 29] analyzed the microbial changes during FAT. Among these, two studies reported the microbial changes of respectively the supragingival [13] and the supragingival plaque in association with saliva [29] that occurred before, during, and after the appliance removal; the third study reported the microbial shift of the subgingival plaque [28] during a short-term observation period (3 months). Two studies assessed the shift of oral microbiome induced by CAT [35]; [3]: one study analyzed the short-term effects (3 months) on the subgingival plaque [35]; the other one evaluated the salivary changes in a long-term period (at least 6 months) [3]. One study reported the microbial differences in change between FAT, CAT, and control groups, through salivary samples.

### 4.5. Interventions Characteristics

Concerning FAT, all patients received vestibular metal brackets. For CAT, two studies analyzed the effects of Invisalign (Align Technology, San Jose, CA, USA) treatments, while in one study the aligner type was not mentioned.

### 4.6. Characteristics of Outcome Measures

**Figure 2:**
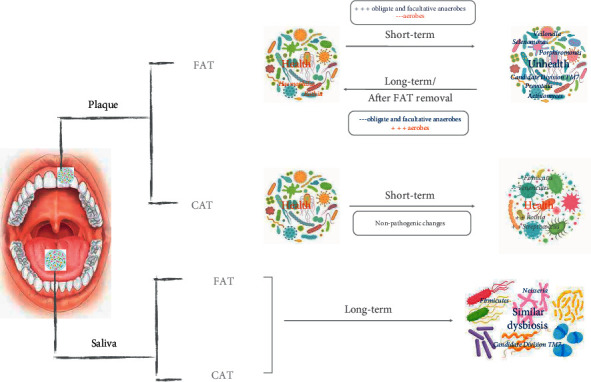
The effects of FAT and CAT on the microbial trends of plaque and saliva.

All studies analyzed the operational taxonomic units (OTUs) abundance and the microbial distribution at phylum, genus, and species level to investigate the shift in microbial community. Core microbiomes and periodontal pathogens (indicators of periodontal disease) were also analyzed. The measured outcomes focused on microbial trends during short term (1–3 months) or long term (at least 6 months) or/and after the end of orthodontic therapy, as shown in Figure 2. Koopman et al. [13] analyzed the microbial changes in time of the supragingival plaque during FAT in adolescents. Even if the microbial diversity increased during the first 6 weeks (*P* < 0.05) and decreased immediately after debonding (*P* < 0.05), no observable shift in the composition of the total community in time was found. A significant change in abundance (*P* < 0.05) was observed only for a few genera: periodontal pathogens (e.g., *Porphyromonas*, *Selenomonas*, *Prevotella*, and *Actinomyces*) became higher in abundance (*P* < 0.05) during the advancement of the orthodontic treatment; the health-related bacteria (*Streptococcus*, *Rothia*, and *Haemophilus*) increased in abundance (*P* < 0.05) towards the end and after orthodontic treatment. Only minor compositional changes remained on the oral microbiome after the end of treatment. Guo et al. [35] focused on CAT short-terms effects on the subgingival microbial community. A slight decreasing alpha microbial diversity (*P* > 0.05) with a significant (*P* < 0.05) change of microbial community (beta diversities were significantly higher after 1 and 3 months) was found during the first three months. The relative abundance of the most periodontal pathogens showed no significant changes (*P* > 0.05) at phylum, genus, and species level. However, significant differences in abundance (*P* < 0.05) were found in several microorganisms across time-points. At phylum level, the relative abundance of the phyla *Firmicutes* and *Tenericutes* was significantly higher at T0 compared with T2 (*P* < 0.05). The phylum *Actinobacteria* showed a slight increase in abundance in time (*P* > 0.05). At genus level, *Mycoplasma* and *Bergeyella* significantly decreased (*P* < 0.05) at T2 and T1, respectively. Additionally, there was no significant difference in the relative abundance of the eight major periodontal pathogens at the genus (*Porphyromonas*, *Tannerella*, *Treponema*, *Campylobacter*, *Prevotella*, *Fusobacterium*, *Capnocytophaga*, and *Veillonella*) and at species (Aa, Pi, Cr, Fn, and Td) levels (*P* > 0.05). Among core microorganisms, genera *Rothia* and *Actinomyces* were higher at T2, genus *Streptococcus* was lower, but without statistically significant differences (*P* > 0.05). Guo et al. [28] analyzed the FAT short-term effects on subgingival microbiome. The alpha diversity indices were stable, while the variation in beta diversity at three different time-points was significantly higher after 3 months. At phylum level, no significant differences in abundance of the predominant phyla (*Firmicutes*, *Bacteroidetes*, *Actinobacteria*, *Proteobacteria*, and *Fusobacteria*) were found (*P* > 0.05). At genus level, the relative abundance of core microbiome was relatively stable. *Actinobacillus* and *Capnocytophaga* showed a temporary increase from T0 to T1 (*P* < 0.05) and a decrease at T2. The abundance of *Granulicatella* significantly reduced between T0 and T2 (*P* < 0.05). At species level, periodontal pathogens showed a temporary increase in relative abundance: *Pi* and *Cr* increased at T1 but returned to baseline at T2; *Fn* and *Td* showed a slight increase at T2, but these changes were not significant. Only *Streptococcus tigurinus* (St) significantly changed, decreasing from T0 to T2 (*P* < 0.05). Wang et al. [34] analyzed the different long-term effects (at least 6 months) between FAT (original F in the paper), CAT (I in the paper) and control (C in the paper) groups. The CAT group was not significantly different from the FAT group, although the abundance of some phyla and genera differed. Among predominant phyla, *Firmicutes* was significantly higher in FAT group (*P* < 0.05), while CAT group did not show a significant increase compared to C group (*P* > 0.05). *Bacteroidetes* showed the lower abundance both in CAT and F group, but only FAT group significantly differed from CAT group (*P* < 0.05). The abundance of candidate division TM7 was significantly higher in CAT rather than in the FAT group (*P* < 0.05). At genus level, *Neisseria* was more abundant in CAT than FAT group (*P* < 0.05) and C group (not significant). The abundance of *Prevotella* and *Fusobacterium* showed higher abundance in C group, although a significant difference was detected only in the FAT group. Among *Rothia* genus, the lower abundance was shown in group C rather than in both groups CAT and FAT, although a significant difference (*P* < 0.05) was shown only between C and FAT groups, which showed the higher abundance. Zhao et al. [36] analyzed 6-month effects of CAT on salivary bacterial community. No significant changes in biodiversity (alpha and beta diversity) were detected in time. At phylum level, significant changes in abundance of the six predominant phyla were not observed, although the increase of *Firmicutes*, *Proteobacteria*, *Bacteroidetes*, and *Actinobacteria* and the decrease of *Fusobacteria* and candidate division TM7. Among genera, there were not any differences in the predominant seven genera among the two groups, except for the increase of *Bacillus* abundance and the decrease of *Prevotella* abundance after 6 months (*P* < 0.05). At the species level, only the decrease in Prevotella_pallens_ATCC_700821 detection was statistically significant (*P* < 0.05).

Kado et al. [29] analyzed FAT long-term effects, through the analysis of supragingival plaque and salivary samples. The diversity analysis indicated dynamic changes of the oral microbiome, more evident in supragingival samples. At phylum level, in plaque samples *Proteobacteria* and *Actinobacteria* decreased and *Bacteroidetes* and TM7 increased after 6 months (*P* < 0.05); in salivary samples, *Actinobacteria*, TM7, and *Spirochaetes* showed significant increase (*P* < 0.05) in time (after 6 months and after removal), while the increase of *Bacteroidetes* and *Fusobacteria* was not significant. At genus level, most of the bacteria increasing in supragingival plaque were obligate anaerobes (as periodontal pathogens *Prevotella*, *Porphyromonas*, *Capnocytophaga*, *Parvimonas*, and *Selenomonas* spp.), while aerobes or facultative anaerobes (as *Actinomyces*, *Corynebacterium*, *Rothia* and *Neisseria*, *Haemophilus*, *Lautropia*) significantly decreased after 6 months. In salivary samples, periodontal pathogens that significantly increased were facultative or obligate anaerobes (as *Prevotella*, *Porphyromonas*, *Capnocytophaga*, *Tannerella*, *Fusobacterium*, *Selenomonas*, and *Atopobium* spp.), while aerobes or facultative anaerobes (as *Neisseria*) decreased with time. *Streptococcus* decreased in supragingival and salivary plaque, but not significantly (*P* > 0.05).

## 5. Discussion

Sufficient evidence reported that orthodontic treatment induces periodontal complications [37, 38], although removable CAT proved to be better for periodontal health and oral hygiene than traditional FAT [1, 6]. Although several studies [1, 39] investigated the differences of clinical parameters, only few of them focused on the microbiological aspects. Orthodontic treatment induces large and rapid changes in composition and activity of the oral community, both temporally and spatially [5, 40]. Therefore, the shift analysis of the entire microbial community during treatment, focusing not only on specific types of bacteria, is fundamental to understand the differences in changes of microbial composition related to these different appliances. Using 16S high-throughput sequencing, all studies reported in this review analyzed saliva and dental plaques (subgingival or supragingival), because they are the two major sources that reflect the oral microbial community [36]. The analysis of microbial diversity was used to evaluate stability and health status of the microbial community [36]. The alpha diversity indices, which reflect microbial evenness and richness in each sample, were relatively stable during early stages of FAT and CAT: a decreasing trend was found for CAT in Guo et al. [35], even if Koopman et al. [13] found a transitional increase of microbial diversity during the FAT first stage that decreased immediately after debonding. In the long-term period, both Zhao et al. [36] and Kado et al. [29] concluded that FAT and CAT do not induce significant changes in salivary biodiversity, even if in supragingival plaque a slight bacterial diversity was found after 6 months [29]. The beta diversity indices represent the variation of microbial communities between samples [29]: the stability of microbial community is connected to periodontal health [41]. According to Guo et al. [28, 35], the beta diversity analysis showed that, in the early stages of FAT or CAT, there was a significant change of microbial composition in subgingival plaque. During FAT, even if Koopman et al. found no shift in beta diversity of the supragingival community in time, Kado et al. [29] detected long-term changes in the composition of oral microbiome during treatment, but only in supragingival plaque and not in salivary samples. Also for CAT, no shift in salivary community diversity was reported in the long-term period [36]. In Koopman et al.'s work [13], the main supragingival microbial periodontal pathogens (belonging to the genera *Veillonella*, *Porphyromonas*, *Selenomonas*, *Prevotella*, and *Actinomyces*) were the highest in abundance during FAT, and most of them decrease in abundance after appliance removal. The members of the genus *Prevotella* include Gram-negative rod-shaped anaerobic bacteria that are associated with the unhealthy state of the periodontium [42]. Although several previous studies have correlated FAT to the increase of *Prevotella intermedia* (Pi) [43], Guo et al. [28] reported a transitional early increase of the species *Pi* in the subgingival plaque, that returned to pretreatment value after 3 months of FAT. This did not coincide with the results of Koopman et al. [13] that, in supragingival plaque, found an increase in abundance in time of the genus *Prevotella* that decreased after the end of treatment, according to van Gastel et al. [43]. Similar microbial results in supragingival plaque were found by Kado et al. [29]: in addition to the significant decrease of the phyla *Proteobacteria* and *Actinobacteria* after six months of FAT as previously reported [13], the genera *Prevotella*, *Porphyromonas*, *Capnocytophaga*, *Parvimonas*, and Selenomonas spp., which are implicated in periodontal disease, significantly increased. So, regarding FAT long-term effects, also Kado et al. [29] reported a significant increase in time of the periodontal pathogens (as *P. gingivalis* and *P. intermedia*) in supragingival plaque that was not transitory [28]. During FAT, both the periodontal genus *Campylobacter* and OTU151 (*Campylobacter gracilis*) decreased with time in supragingival plaque [13]. A similar pattern of decrease of *Campylobacter rectus* (Cr) has been reported in the subgingival plaque by previous literature [44, 45]: also Guo et al. [28] reported that the periodontal species of *Cr* transiently increased after 1 month, then returned to baseline level after three months. During early stages, the FAT influenced the subgingival plaque microbial community, as reported by Guo et al. [28]: these data are in accordance with short-term results of Koopman et al. [13] that reported a significant microbial change of supragingival plaque after 6 weeks. Among potential periodontal pathogens, Guo et al. [28] reported a transient increase of genus *Capnocytophaga* that was related to the decrease of genus *Granulicatella* and of the species *S*. *tigurinus*, a novel pathogen causing endocarditis, meningitis and periodontal disease [46, 47]. Among periodontal core microbiome, according to Koopman et al. [13], in Guo et al.'s work [28], the genus *Veillonella* was stable, while the genus *Actinobacillus* showed a temporary increase in the first month, decreasing in time; the candidate division TM7 (TM7), a core microbiome related to gingivitis and periodontitis [13, 48, 49] showed no significant changes during the first three months of FAT, contrary to the decrease of TM7 (and OTUs 55, 171, 355) reported by Koopman et al. [13] that could be related to the advancing age of the study sample [50]. So, as reported by Guo et al. [28], the relative stability of core microbiome and of the periodontal species (*Pi*; *Cr*; *Fusobacterium nucleatum*, *Fn*; *Treponema denticola*, *Td*) during the first three months of FAT might represent a relatively healthy periodontal status and might suggest a risk of only transient periodontal inflammation in the early stages of FAT [28]. After the end of FAT, Koopman et al. [13] observed a reduction in time for the genera *Porphyromonas* and *Selenomonas* and for OUT302 (*Selenomonas*) that included the main periodontal pathogens [49, 51]: these favorable changes could be explained by the reduction of retention sites due to the alignment of teeth or by the fixed appliance removal [13]. In fact, towards the end of treatment and after appliance removal, *Haemophilus*, *Rothia*, and OTU65 (*Rothia*), which are often associated with health status [52], showed an increasing trend: also the abundance of the genus *Streptococcus* and OUT351 (*Streptococcus*) increased in time, suggesting that FAT induces minimal changes in the oral microbial composition and do not negatively affect oral health, if oral hygiene is properly maintained [13]. Furthermore, a previous review [33] indicated that FAT might not permanently induce periodontal disease by affecting the subgingival periodontal pathogen levels, temporarily increasing after appliance placement and returning to pretreatment level several months later. Kado et al. [29] reported that, both in saliva and supragingival plaque, the bacteria that significantly increased are obligate and facultative anaerobes, and that the relative abundance of aerobes decreased during FAT. So, these data suggest that FAT alters the oral microbiome (especially in plaque) towards a pathogenic state of periodontium. Many genera of actinobacteria (as *Actinomyces*, *Corynebacterium*, and *Rothia*) and of proteobacteria (as *Neisseria*, *Haemophilus*, and *Lautropia*) showed a significant decrease after 6 months. These results are in contrast with Koopman et al. [13] that reported an increase of *Rothia*, *Neisseria*, and *Haemophilus* in supragingival plaque during the advancement of FAT. During FAT long-term effects, in saliva, the bacteria that increased in time were facultative or obligate anaerobes (as *Prevotella*, *Porphyromonas*, *Capnocytophaga*, *Tannerella*, *Fusobacterium*, *Selenomonas*, and *Atopobium* spp.), while the changes in health-related bacteria (as *Neisseria* and *Streptococcus*) were stable. The phylum *Saccharibacteria* (formally TM7), detected only by NGS technology and not by laboratory culture, showed a significant abundance increase in time, both in supragingival plaque and saliva [29], according to the short-term increase in subgingival plaque reported by Guo et al. [28]. So, Kado et al. [29] suggest that FAT induces a transitional shift of microbiome from healthy to periodontitis, especially of the gingival microbiome, according to previous literature [33] and to the results of Koopman et al. [13]. During the first three months, Guo et al. [35] demonstrated that, although CAT decreased the microbial diversity and changed the microbial community, it influenced less the core microbiota and periodontal pathogens. While *Actinobacteria* showed an increased trend in abundance, the phyla *Firmicutes* and *Tenericutes* significantly decreased in abundance at the early stages of CAT, while at the genus level, a reduction of *Mycoplasma* and *Bergeyella* was found, after, respectively, 1 month and 3 months [35]: the authors suggest that the reduction of the phylum *Firmicutes* and of the genus *Mycoplasma*, that are involved respectively in periodontitis [53] and gingivitis [54], could be associated to a better oral hygiene related to CAT. In addition, no significant differences in relative abundance of the periodontal pathogens (Aa, Pi, Cr, Fn, and Td) at species level were found by Guo et al. [35], even if a slight decrease of Pi and a slight increase of Aa were found during CAT short-term period. The relative stability of core microbiome could be associated to a health periodontal status, the relative abundance of the genera *Rothia* and *Streptococcus*, both nonpathogenic microorganism in periodontal disease [55], were higher after three months of CAT. So, the author suggested that CAT, although it influenced the microbial community, did not induce pathogenic changes of the subgingival microbiome during the first three months [35]. Contrary to FAT [29], during long-term observation time, Zhao et al. [36] reported that CAT also induced a significant decrease of the genus *Prevotella* (and of the species *Prevotella pallens*) at salivary level that, in association with a significant increase of the *Bacillus*, indicates a healthier oral condition with less risk of periodontitis. At phylum level, the reduction of *Fusobacterium* and candidate division TM7 was not significant, as the increase of pathogens *Firmicutes*, *Bacteroidetes*, *Actinobacteria*, and *Proteobacteria* [36]: so, Zhao et al. [36] concluded that CAT does not induce significant biodiversity changes in microbial community and does not worsen periodontal health, if patients have better oral hygiene habits during treatment. Wang et al. [34] investigated the influence of FAT and CAT on the oral microbiome, compared to controls. They found significant differences of *Firmicutes* and TM7 between FAT, CAT, and C groups. Compared to controls, FAT induces a long-term increase of Firmicutes, even if Koopman et al. [13] and Guo et al. [28] showed no significant differences in short-term observation time. In CAT group, the abundance of *Firmicutes* was less than FAT group and similar to controls, according to the salivary results reported by Zhao et al. [36]. Instead, Guo et al. [35] reported a significant decrease of *Firmicutes* in the first three months of CAT. The candidate division TM7 was significantly higher in CAT group than FAT group, in opposition to the previous long-term results in which TM7 showed no significant changes during CAT [3] and significantly increased after FAT [29]. So, these data suggest that CAT induces more periodontal complications associated to TM7. Also, genus *Neisseria* was more abundant after CAT than FAT, while a decreasing abundance tendency was found in the FAT group, according to Koopman et al. [13] and Kado et al. [29], compared to controls. *Prevotella* genus showed a significant decrease in FAT compared to controls, even if previous studies reported a significant increase in the long term [13, 29]. So, Wang et al. [34] suggest that both CAT and FAT induce dysbiosis of the oral microbiome and that, regarding microbial composition and diversity, CAT did not show better performance compared to FAT, in disagreement with previous studies [1, 6] in which CAT improved periodontal health. Probably these data were not related to better oral conditions but to implemented oral hygiene measures [34].

The development of new molecular technologies, as NGS, has provided a key contribution to the oral microbiome recent knowledge. In the molecular microbiology field, the monitoring of the microbial alterations in the oral cavity is essential in order to identify the oral microbial shift induced by two different orthodontic therapies (FAT and CAT). The knowledge of the complex changes induced by orthodontic therapies in oral microbiome in terms of quantity and quality [20] may be an indirect way to follow the clinical changes in local and systemic health parameters for evaluating the risk of development or deterioration of existing conditions, as local or systemic complications [17]. The imbalance of microbial flora contributes to oral and systemic diseases [15]; therefore, the microbiological findings of this review may be indeed clinically translated to support patient care, in terms of prevention, monitoring, or therapeutic choice [17]. These preliminary findings suggest that FAT induces a higher level of pathogenic periodontal bacteria compared to CAT, but these pathological changes are mainly at an early stage and located in subgingival plaque, because no long-term differences at salivary level were found for both appliances. Thus, the risk for patients of developing dental or periodontal disease with FAT could happen at an early stage because this pathological shift is transient in the presence of proper oral hygiene. Through these microbial findings, the clinicians may decide the most appropriate and individualized treatment based on the patient's clinical conditions, especially in susceptible patients with high risk of local or systemic complications. [17]. However, further studies are needed to investigate these topics.

## 6. Conclusions

According to SBU tool, this review could draw conclusions with a limited level of evidence.

At supragingival level, FAT seems to determine a transient change of microbial biodiversity and composition that returns to baseline after the end of the therapy: studies about CAT effects have not been performed yet.

At subgingival level, FAT and CAT seem to induce a transitional significant change in the composition of microbiome that maintains a relatively stable biodiversity: during early stages, FAT induces a slight increase of periodontal subgingival pathogens while CAT seems to induce no pathogenic changes of subgingival microbiome. At salivary level, FAT and CAT do not seem to affect microbial composition and biodiversity: during the long-term period, the dysbiosis of salivary microbiome was similar for both appliances.

Considering the small size of included studies, the relatively short observation time, the clinical heterogeneity of the studies, and the different sample site collection, this review reflects only the changing trend in the oral microbial community during orthodontic treatment. Further high-quality randomized clinical trials with accurate methodologies and appropriate sample size are needed to increase the quality of evidence about microbial changes during orthodontic treatment.

## Figures and Tables

**Table 1:** Characteristics of the included studies.

Author/year	Study design	Sample size/groups	Average age/age range (y)	Sample site	Orthodontic appliance	Sample collection time	Collection method	Microbial analysis method	Taxonomic analysis	Microbial outcome	Quality of the study
Koopman et al. [13]	Randomized controlled clinical trial	91 Group 1 (fixed): with mouthwash Group 2 (fixed): without mouthwash	10–16.8	Supragingival plaque of 2.4 + 2.5	Fixed	T0: 1 week before placement; T1: 6 weeks after placement; T2: 12 weeks after placement; TD: at debonding (average 25 months after placement) TD1: 6 weeks after debonding; TD2: 12 weeks after debonding	Sterile plastic spatula	16S rRNA sequencing (V5–V7 regions)	15 phyla 149 genera 461 OTUs	Minimal changes after the end of treatment (if oral hygiene is maintained)	B

Guo et al. [33]	Prospective study	10 (10 F)	25.4 ± 6.2	Subgingival plaque of 11 + 21, 31–41, 16 + 26, 36–46	Clear aligners	T0: before treatment T1: after 1 month T2: after 3 months	Sterile curette	16S rRNA sequencing (V3–V4 regions)	15 phyla 85 genera 5 species analyzed (Aa, Pi, Cr, Fn, Td) 414 OTUs	Not early pathogenic changes	B

Guo et al. [28]	Prospective study	10 (10 F)	18–40	Subgingival plaque of 11 + 21, 31–41, 16 + 26, 36–46	Fixed	T0: before treatment T1: after 1 month T2: after 3 months	Sterile curette	16S rRNA sequencing (V3–V4 regions)	12 phyla 105 genera 5 species analyzed (Pi, Cr, Fn, Td, St) 417 OTUs	(Transient) early shift to mild inflammation	B

Wang et al. [34]	Prospective study	15 Group 1 (fixed): 5 Group 2 (clear aligner): 5 Group 3 (control): 5	20–25y	Salivary samples	Fixed Clear aligners (Invisalign)	T0: before appliance placement T1: after 6 months	Not mentioned	16S rRNA sequencing (V3–V4 regions)	21 phyla 124 genera 260.3 OTUs	Long-term dysbiosis for both appliances	B

Zhao et al. [3]	Prospective study	25 (22 F, 3 M)	20–35y	Salivary samples	Clear aligners (Invisalign)	T0: before treatment T1: after 6 months	Sterile cryogenic vials	16S rRNA sequencing (V3–V4 regions)	15 phyla 15 genera 10 species analyzed 8,885 OTUs	No long-term changes	B

Kado et al. [29]	Prospective study	71	21.1 ± 7.4	Supragingival plaque of upper and lower anterior teeth + salivary samples	Fixed	For supragingival plaque: T0: before treatment T1: after 6 months For salivary samples: T0: before treatment T1: after 6 months T2: after removal	Sterilized explorer + clean paper cup	16S rRNA sequencing (V1–V2 regions)	10 phyla 31 families 44 genera 33 species analyzed 983 OTUs	Transitional microbial shift to periodontitis	B

F = female; M = male; OTUS = operational taxonomic units; V1–V2; V3–V4; V5–V7 = amplified regions of the bacterial 16S gene; Aa = *Aggregatibacter actinomycetemcomitans*; Pi = *Prevotella intermedia*; Cr = *Campylobacter rectus*; Fn = *Fusobacterium nucleatum*; Td = *Treponema denticola*; St = *Streptococcus tigurinus*; Pp = *Prevotella pallens*; Ag=*Actinomyces graevenitzii*; Sp = *Streptococcus parasanguis*; L = *Leptotrichia*.

## Data Availability

Data sharing is not applicable to this article as no new data were created or analyzed in this study.
